# Association between metabolic and hormonal profile, proinflammatory cytokines in saliva and gingival health in adolescent females with polycystic ovary syndrome

**DOI:** 10.1186/s12903-021-01553-9

**Published:** 2021-04-13

**Authors:** Natalia Wendland, Justyna Opydo-Szymaczek, Dorota Formanowicz, Anna Blacha, Grażyna Jarząbek-Bielecka, Małgorzata Mizgier

**Affiliations:** 1 Chair and Department of Pediatric Dentistry, Poznan University of Medical Sciences, 70 Bukowska Street, 60-812 Poznan, Poland; 2 Chair and Department of Medical Chemistry and Laboratory Medicine, Poznan University of Medical Sciences, 8 Rokietnicka Street, 60-806 Poznan, Poland; 3 Department of Perinatology and Gynecology, Division of Developmental Gynecology and Sexology, Poznan University of Medical Sciences, 22 Polna Street, 60-535 Poznan, Poland; 4 Department of Dietetics, Faculty of Physical Culture in Gorzów Wielkopolski, Poznan University of Physical Education, 13 Estkowskiego Street, 66-400 Gorzów Wielkopolski, Poland

**Keywords:** Polycystic ovary syndrome, Gingivitis, Body Mass Index, Hyperandrogenism, Testosterone, Lipid profile, Insulin resistance, Salivary cytokines

## Abstract

**Background:**

Research studies indicate that polycystic ovary syndrome (PCOS) may increase susceptibility to periodontal disease. The mechanisms that link both conditions are not entirely understood. Thus, the study aimed to investigate the impact of hormonal and metabolic disturbances on the gingival health and salivary levels of tumor necrosis factor (TNF-α), interleukin 1β (IL1-β), and interleukin 6 (IL-6) in adolescent girls with PCOS.

**Methods:**

Thirty-one patients with PCOS and twenty-eight healthy age-mates (as the control group) were enrolled in the study. Individuals with PCOS underwent blood tests for the determination of hormonal and metabolic parameters. Saliva samples were collected to measure salivary testosterone and proinflammatory cytokines in both studied groups. Calibrated dentist assessed oral hygiene and gingival health of all subjects.

**Results:**

Salivary testosterone was significantly higher in the study group (*p* = 0.0007). The groups did not differ significantly concerning periodontal parameters. Patients with PCOS revealed higher levels of salivary cytokines (*p* < 0.0001). Gingival index (GI) and the percentage of sites bleeding upon probing (BOP%) were positively correlated with the plaque index (PI) in both groups (r_s_ ≥ 0.60, *p* < 0.001), and negatively correlated with salivary testosterone level in the PCOS group (r_s_ = − 0.44, *p* = 0.0138 and r_s_ = − 0.37, *p* = 0.0424, respectively). BOP% was also positively correlated with body mass index (BMI) in the control group (r_s_ = 0.40, *p* = 0.0368) and index of insulin resistance (HOMA-IR) in the study group (r_s_ = 0.48, *p* = 0.0068). Salivary testosterone was positively correlated with TNF-α in the control group (r_s_ = 0.41, *p* = 0.0321), while in the study group, total testosterone (TT) was positively correlated with IL-6 (r_s_ = 0.37, *p* = 0.0400) and free androgen index (FAI) with TNF-α (r_s_ = 0.36, *p* = 0.0491).

**Conclusions:**

Gingival health of the examined population was associated primarily with oral hygiene and, to a lesser extent, with the hormonal and metabolic profile. Despite similar periodontal parameters in the both studied groups, patients with PCOS revealed significantly higher levels of proinflammatory cytokines in saliva, which might be the manifestation of the systemic low-grade inflammation associated with PCOS.

## Background

Polycystic ovary syndrome (PCOS) is one of the most frequently diagnosed endocrinopathies that affects 7–20% of women of reproductive age [1]. This complex condition’s main symptoms are menstrual disorders, hyperandrogenism, coexisting obesity, insulin resistance, and abnormal lipid profile in some women. PCOS is the most common cause of female infertility, increases the risk of cardiovascular diseases, type 2 diabetes, endometrial cancer, and psychological problems such as depression, anxiety, and eating disorders [1–3]. The most common reason for implementing diagnostics to confirm or exclude PCOS is clinical hyperandrogenism, i.e. a state of increased androgens' production in the body. Hyperandrogenism manifests itself by menstrual cycle disorders, lack of ovulation, and the appearance of male characteristics: masculinization, acne, seborrhea, changes in body proportions, voice color, and male hair type [1, 2].

Reports from the literature indicate that PCOS is not only associated with infertility and complications of metabolic syndrome but may also increase the risk of periodontitis [4–10]. The mechanisms that link PCOS and periodontal problems are not fully explained. Results of some studies indicate that PCOS may affect the oral microflora composition [6, 11], but the evidence is equivocal [12]. On the other hand, the hormonal and metabolic disturbances associated with PCOS might increase host's susceptibility to periodontal disease [10].

It has been reported that women with PCOS have increased serum levels of inflammatory markers, including CRP and pro-inflammatory cytokines, like tumor necrosis factor (TNF-α), interleukins (IL): IL-1, and IL-6, compared to healthy control patients [13–15]. Proinflammatory cytokines are also elevated in patients suffering from periodontal disease [16]. Various studies suggest the involvement of TNF-α, IL-1, and IL-6 in the development of insulin resistance, which is a typical metabolic trait of PCOS [14, 17]. These factors lead to the activation of signaling pathways that impair insulin signaling [17]. Compensatory hyperinsulinemia in women with endocrinopathy contributes to androgen-dependent ovarian dysfunction [18]. In the view of these data, periodontal disease in women with PCOS may potentially exacerbate its clinical characteristics [10]. A recent randomized clinical trial showed that non-surgical periodontal treatment and a myo-inositol supplementation significantly reduced CRP levels and insulin resistance [19].

Most studies evaluating the association between PCOS and periodontal health have been carried out in adults [4–11], while the assessment of factors affecting periodontium in adolescence may help to prepare guidelines for the prevention of irreversible periodontitis in mature females.

Thus, the study aimed to investigate the impact of hormonal and metabolic disturbances, on the gingival health and salivary concentrations of TNF-α, IL-1β, and IL-6 in adolescent girls with PCOS.

We have hypothesized that young females with PCOS do not differ from healthy controls concerning gingival parameters, but some hormonal and metabolic traits of the endocrinopathy might be associated with periodontal problems in this vulnerable group of patients. Secondly, we assumed that salivary cytokine levels in females with PCOS are higher than in the control group due to chronic low-grade inflammation associated with the disease.

## Methods

The study protocol was approved by the University Bioethics Committee (Resolution No. 536/18), and the written informed permission was provided by all participants and parents of subjects younger than 18 years.

### Study population

The study group consisted of 31 adolescent females aged 15–19 years. The participants were recruited at the Gynecology and Obstetrics Hospital of Poznan University of Medical Sciences (Poland, Wielkopolska voivodeship). All subjects were at least two years after menarche and had newly diagnosed, not previously treated PCOS. Inclusion to the group was based on the Rotterdam criteria [1, 2], i.e. the presence of at least two of the following: polycystic ovaries on ultrasound, biochemical or clinical hyperandrogenism [20–22], oligo-ovulation (based on oligomenorrhea or secondary amenorrhea).

We excluded patients with any other systemic diseases including obesity, thyroid disorders, Cushing syndrome, androgen-secreting tumors, congenital adrenal hyperplasia, prolactin excess suggestive of pituitary adenoma, hormonal therapy, using orthodontic appliances or antibacterial mouthwashes, reporting smoking, having complications of dental caries (clinical signs of periapical inflammation), receiving antibiotics during the last six months.

The control group of 28 healthy females aged 15–19 was recruited at the University Center of Stomatology and Specialist Medicine in Poznan based on the oral health screening. They were matched concerning oral hygiene and age to the PCOS subjects.

We excluded patients with systemic diseases including obesity, having symptoms of possible hormonal disturbances such as irregular periods, amenorrhea, oligomenorrhea, short or heavy menstrual bleedings, moderate or severe acne according to Investigator’s Global Assessment scale, hirsutism (> 7 points on the modified Ferriman—Gallwey scale) [20, 21], reporting smoking, having complications of dental caries (clinical signs of periapical inflammation), using orthodontic appliances or antibacterial mouthwashes, receiving antibiotics during the last six months.

### Medical evaluation

The basic anthropometric measurements in the study group were taken on admission to the hospital. Diagnosis of excessive weight was based on BMI and the WHO growth reference medians for children aged 5–19 years [22].

Gynecological examination and blood tests were carried out in the early follicular phase, apart from one patient with secondary amenorrhea.

The blood samples were collected in the morning on fasting, and additionally in the evening to reassess cortisol levels. Laboratory evaluations included: luteinizing hormone (LH), follicle-stimulating hormone (FSH), estradiol, total testosterone (TT), dehydroepiandrosterone sulfate (DHEA-S), sex hormone-binding globulin (SHBG), cortisol, fasting glucose, fasting insulin, triglycerides (TG), total cholesterol (TC), and high-density lipoprotein cholesterol (HDL-C).

After two days spent at the hospital, the patients were referred to the University Center of Stomatology and Specialist Medicine for clinical periodontal evaluation.

The control group of subjects recruited in the University Center of Stomatology and Specialist Medicine was referred for the gynecological consultation in the hospital outpatient clinic. The gynecologist (G.J.-B.) verified the presence of symptoms and signs of androgen excess in the control group. Participants answered questions about menstrual cycle regularity*.* Physical examination included: measurement of height and weight, evaluation of skin for signs of hirsutism and acne. Due to ethical concerns about unnecessary stressful procedures in children, blood tests and gynecological examination were carried out only in patients with PCOS. Healthy subjects who qualified for the study were further periodontally evaluated in the University Center of Stomatology and Specialist Medicine.

### Dental examination

The oral hygiene was assessed according to the Silness-Löe plaque index (PI) and the gingival health with the use of the Gingival Index (GI) on the six index teeth: 16, 12, 24, 36, 32, 44. The subject's oral hygiene was described as follows: poor (PI = 1.9–3.0), average (PI = 0.7–1.8), good (PI = 0–0.6). The gingival health was assigned as follows: healthy (GI = 0), mild gingivitis (GI = 0.1–1), moderate gingivitis (GI = 1.1–2), severe gingivitis (GI = 2.1–3) [23, 24]. The probing depth (PD) measurements were performed using a Williams periodontal probe (Hu-Friedy Mfg. Co. LLC, Chicago, USA). Bleeding upon probing score (BOP%) was assessed as the percentage of sites bleeding after stimulation with a probe. The measurements of PD and BOP% were recorded on all teeth at six sites (buccal, distobuccal, mesiobuccal, lingual, distolingual, and mesiolingual) [25].

All subjects were examined by a calibrated pediatric dentist (N.W.). Calibration exercises were performed on healthy teenage patients, not included in the main study. The examiner was considered calibrated when she reached substantial correlation of repeated measurements and a considerable correlation with evaluations of her trainer (J.O.-S) (Cohen’s Kappa > 80).

### Saliva sampling

Patients were asked to abstain from eating and drinking for 2 h before saliva sampling. Unstimulated whole mixed saliva was collected using the Salivette® (Sarstedt Laboratories; Germany) system. The saliva sample specimens were centrifuged at 4000 rpm for 10 min, then aliquoted and stored at − 80 °C until carrying out the assays.

### Biochemical parameters

Biochemical analyses of blood samples were carried out in the hospital laboratory. TC, HDL-C, TG levels were determined by the enzymatic colorimetric method (Roche Diagnostics GmbH, Mannheim, Germany). Low-density lipoprotein cholesterol (LDL-C) was calculated with the use of Friedewald’s formula (LDL-C [mg/dl] = TC-HDL-C-TG/5). Fasting blood glucose was measured by the enzymatic method with hexokinase. Insulin, LH, FSH, TT, 17-β-estradiol, DHEA-S, cortisol, SHBG were measured by electrochemiluminescence (ECLIA) immunoassay method (Elecsys) (Roche Diagnostics Gmbh, Mannheim, Germany). Free androgen index (FAI) was calculated based on TT and SHBG levels using the online calculator (https://www.siemens-healthineers.com). Homeostasis model assessment of insulin resistance (HOMA-IR) was calculated from fasting glucose levels and fasting insulin [26]. Obtained results were compared to the sex and age-specific laboratory reference values and the literature data [26–29].

Analyses of inflammatory biomarkers and salivary testosterone levels were performed by medical lab analyst (A.B.) in the Chair and Department of Medical Chemistry and Laboratory Medicine laboratory, Poznan University of Medical Sciences. Salivary concentrations of the inflammatory markers (IL-6, IL-1β, TNF-α) were measured with a commercial enzyme-linked immunosorbent assay kits purchased from Shanghai Sunred Biological Technology Co. (China) and testosterone level was measured with a commercial enzyme-linked immunosorbent assay kit purchased from DRG International Inc. (USA) using the TECAN-SUNRISE reader with the Magellan software.

### Statistical analysis

The sample size calculation was performed with the G*Power 3.1.9.2 software. Based on the report of Dursun et al. [8] concerning young females suffering from PCOS, we expected a large effect size (d > 0.80). A difference of 0.50 in the GI and SD of 0.6 were considered as reference values. To achieve a power of 0.80 with α set at 0.05, minimum 20 subjects were required in each group to detect the expected difference with the use of non-parametric Mann–Whitney U test.

Data were analyzed using the Statistica software version 12 (StatSoft. Inc. 2014, Tulsa, USA) with significance taken as *p* < 0.05. The Mann–Whitney U test was used to evaluate differences between groups, while Spearman’s rank test for analyses of correlation between quantitative data. We did not use Bonferroni adjustment for multiple tests, because our study was explanatory in nature and this conservative approach would increase the probability of a type II error.

## Results

Table 1 presents subjects’ characteristics, including age, BMI, oral hygiene, and periodontal parameters.

**Table 1:** Characteristics of the studied groups including age, BMI, oral hygiene, and gingival health

	Group	No. of subjects	Mean	Median	Min	Max	SD	Reference value	No. of subjects with abnormal values
Age	PCOS	31	16.5	17.0	15.0	19.0	1.2	N.A	N.A
	Control	28	16.1	16.0	15.0	19.0	1.2		
BMI	PCOS	31	22.4^1^	22.4	16.0	27.7	3.4	< 1SD of the reference median [22]	10↑
	Control	28	20.6	20.0	16.2	26.9	3.1		5↑
PI	PCOS	31	0.72	0.75	0.08	1.66	0.45	< 0.7 (good hygiene) [23, 24]	↑17
	Control	28	0.62	0.61	0.04	1.46	0.46		↑14
GI	PCOS	31	0.39	0.29	0.04	1.29	0.35	≤ 1 mild gingivitis [23, 24]	↑2
	Control	28	0.35	0.25	0.00	1.08	0.32		↑1
BOP%	PCOS	31	12.4	9.2	1.6	45.3	11.6	< 10% gingival health [25]	↑13
	Control	28	13.0	8.5	1.2	33.1	10.6		↑14
PD	PCOS	31	0.83	0.83	0.50	1.00	0.14	≤ 3 [25]	↑0
	Control	28	0.78	0.83	0.50	1.00	0.17		↑0

BMI, body mass index; PI, plaque index; GI, gingival index; BOP%, bleeding on probing score; PD, probing depth; TT, total testosterone, N.A., not applicable

↑ above, ↓below the reference values

^1^*p* = 0.0375 as compared to healthy controls, Mann–Whitney U test

BMI of subjects from both groups differed significantly (22.4 and 20.6, *p* = 0.0375, respectively). There were 10 subjects with overweight in the study group, while in the control group, 5 subjects had BMI above 1SD of the growth reference median.

PI, BOP%, and PD of both groups did not differ significantly (*p* > 0.05). Three patients had moderate gingivitis (GI > 1): two in the study group and one in the control group. Gingivitis, defined as ≥ 10% of sites with bleeding on probing, was noted in 13 (41.9%) subjects with PCOS and 14 (50%) healthy controls.

Table 2 shows salivary testosterone and cytokines levels in both study groups, while Table 3 summarizes serum hormonal and biochemical parameters of the PCOS group.

**Table 2:** Salivary testosterone and cytokines levels in the studied groups

	Group	No. of subjects	Mean	Median	Min	Max	SD
Salivary testosterone (pg/ml)	PCOS	31^a^	40.87	37.18	23.46	69.91	13.39
	Control	28	28.59	29.72	15.60	44.09	7.58
TNF-α (pg/ml)	PCOS	31^b^	21.37	16.51	12.04	69.72	14.61
	Control	28	11.79	11.84	8.12	14.80	1.58
IL-6 (pg/ml)	PCOS	31^b^	11.99	8.97	5.97	42.72	8.77
	Control	28	5.74	5.66	3.00	8.77	1.56
IL-1β (pg/ml)	PCOS	31^b^	177.11	170.32	67.27	394.53	54.24
	Control	28	120.29	125.11	55.81	161.20	25.90

TNF-α, tumor necrosis factor α; IL-6, interleukin 6; IL-1β, interleukin 1β; N.A., not applicable

^a^*p* = 0.0007 as compared to healthy controls, Mann–Whitney U test

^b^*p* < 0.0001 as compared to healthy controls, Mann–Whitney U test

**Table 3:** Serum hormonal and biochemical parameters of the PCOS group

	Mean	Median	Min	Max	SD	Reference value	No. of subjects with abnormal values
FSH (mlU/ml)	4.94	5.18	1.32	9.42	2.01	3.5—12.5*	9↓
LH (mlU/ml)	11.43	8.43	0.20	27.73	7.64	2.4—12.6*	12↑, 3↓
LH/FSH	2.38	2.19	0.15	8.54	1.71	≤ 2 [22]	15↑
estradiol (pg/ml)	66.84	45.44	12.55	236.40	56.71	12.5–166* (folicular phase)	4↑
TT (ng/dl)	53	49	6	118	20	< 51 [23]	14↑
SHBG (nmol/l)	56.86	47.55	20.21	176.10	33.21	26.1–110*	5 ↓ 1↑
FAI	4.32	3.60	0.64	12.00	2.99	≤ 4.4 [21]	10 ↑
DHEA-S (µmol/l)	6.96	5.94	2.17	12.14	2.81	1.77–9.99 *	7↑
cortisol (morning sample) (nmol/l)	395.40	431.70	105.10	575.00	121.57	166–507*	2↓ 6↑
cortisol (evening sample) (nmol/l)	80.21	56.95	15.37	300.70	70.24	74–291*	17↓1↑
fasting glucose (mg/dl)	87.77	87.40	78.80	99.00	5.22	60–99*	0↑
fasting insulin (mU/ml)	14.55	14.76	3.78	31.51	6.37	2.6–24.9*	2↑
HOMA-IR	3.17	3.20	0.34	7.05	1.51	< 2.32 [20]	23↑
TC (mg/dl)	156.91	157.70	120.50	208.10	24.09	< 190*	3↑
HDL-C (mg/dl)	54.82	55.60	38.90	73.20	8.65	≥ 45*	2↓
LDL-C (mg/dl)	83.51	79.50	44.30	130.60	20.71	< 115*	3↑
TG (mg/dl)	92.92	84.50	46.70	201.90	37.46	< 150*	3↑

FSH, follicle-stimulating hormone; LH, luteinizing hormone; IU, international units; TT, total testosterone; FAI, free androgen index; SHGB, sex hormone-binding globulin; DHEA-S, dehydroepiandrosterone sulfate; HOMA-IR, Homeostatic Model Assessment of Insulin Resistance; TC, total cholesterol; HDL-C, high-density lipoprotein cholesterol; LDL-C, low-density lipoprotein cholesterol; TG, triglycerides

^*^According to the hospital age- and sex-specific laboratory reference ranges, ↑above, ↓below the reference values

There were statistically significant differences between concentrations of TNF-α, IL-6, and IL-1β in the saliva of both groups of subjects (*p* < 0.0001). Salivary testosterone level was significantly higher in the study group (*p* = 0.0007).

Regarding oral hygiene procedures, in the study group 26 (83.4%) subjects brushed their teeth at least twice daily, 6 (19.4%) used dental floss, 12 (38.7%) electric or sonic toothbrush, while in the control group, 28 (100%) subjects reported twice daily brushing, 4 (14.3%) reported flossing, and 15 (53.6%) used electric or sonic toothbrush (data not listed in tables).

Among the subjects with PCOS, 3 (9.7%) had polycystic ovarian morphology, 1 (3.2%)—secondary amenorrhea, 30 (96.8%) oligomenorrhea, 27 (87.1%) mild hirsutism (8–16 points on the modified Ferriman–Gallwey scale), 4 (12.9%)—moderate hirsutism (17–25 points on the modified Ferriman–Gallwey scale), 12 (38.7%) severe acne, and 16 (51.6%) moderate acne [20, 21], 15 (48.4%) LH/FSH ratio > 2 [28], 4 (12.9%) estradiol above the reference values, 7 (22.6%) increased levels of DHEA-S, 6 (19.4%) morning cortisol above reference value, 1 (3.2%) afternoon cortisol above reference value, 14 (45.2%) TT > 50 ng/dl [29], and 10 (32.3%) free androgen index above the reference range [27]. Regarding glucose metabolism and lipid profile: 2 (6.5%) subjects had elevated fasting insulin levels, 23 (74.2%) had HOMA-IR index above the cut-off point [26], 3 (9.7%) subjects had elevated levels of TC and LDL-C, 3 (9.7%) had increased TG, and 2 (6.5%) had lowered concentration of HDL-C.

Table 4 presents Spearman’s correlation coefficients for pairwise variables and p-values (p) of statistically significant associations.

**Table 4:** Spearman’s correlation coefficients (r_s_) between periodontal parameters, salivary cytokines and selected factors analyzed in the PCOS group (n = 31) and the control group (n = 28)

	N	GI		BOP%		PD		IL-1β (pg/ml)		IL-6 (pg/ml)		TNF-α (pg/ml)	
		r_s_	p	r_s_	P	r_s_	p	r_s_	p	r_s_	p	rs	p
PI	31	0.61	0.0003*	0.60	0.0004*	0.17	0.3512	− 0.07	0.6947	0.07	0.7259	0.07	0.7153
	28	0.66	0.0002*	0.79	0.0000*	0.26	0.1733	− 0.35	0.0665	0.07	0.7277	− 0,10	0.5970
BMI	31	0.14	0.4101	0.26	0.1568	0.02	0.9029	− 0.17	0.3571	0.07	0.6964	0.18	0.3452
	28	0.36	0.0604	0.40	0.0368*	0.37	0.0497*	− 0.05	0.8151	− 0.17	0.3761	0.07	0.7333
Estradiol (pg/ml)	31	− 0.04	0.8202	0.05	0.7719	0.21	0.2492	− 0.24	0.1903	0.07	0.6980	0.05	0.7894
TT (ng/dl)	31	− 0.34	0.0622	− 0.27	0.1422	− 0.22	0.2417	0.08	0.6683	0.37	0.0400*	0.20	0.2891
FAI	31	− 0.20	0.2783	− 0.11	0.5495	− 0.18	0.3276	0.04	0.8403	0.32	0.0836	0.36	0.0491*
SHBG (nmol/l)	31	− 0.02	0.9164	− 0.15	0.4241	− 0.03	0.8560	− 0.02	0.9117	− 0.15	0.4308	− 0.15	0.4193
DHEA-S (µmol/l)	31	− 0.05	0.7884	− 0.15	0.4165	− 0.17	0.3561	0.33	0.0675	0.06	0.7482	0.26	0.1510
Cortisol (morning sample) (nmol/l)	31	0.14	0.4382	0.18	0.3333	0.08	0.6837	0.12	0.5308	0.30	0.1050	− 0.08	0.6852
Cortisol/DHEA-S	31	0.11	0.5609	0.30	0.0999	0.22	0.2265	− 0.09	0.6303	0.18	0.3397	− 0.18	0.3412
Fasting glucose (mg/dl)	31	− 0.04	0.8219	− 0.13	0.4844	− 0.27	0.1463	0.07	0.6979	0.05	0.7852	0.15	0.4104
Fasting insulin (mU/ml)	31	0.23	0.2044	0.36	0.0469*	0.21	0.2669	0.08	0.6780	0.24	0.2022	0.01	0.9716
HOMA-IR	31	0.28	0.1301	0.48	0.0068*	0.24	0.1866	0.11	0.5535	0.14	0.4456	0.04	0.8202
TC (mg/dl)	31	− 0.31	0.0869	− 0.06	0.7325	− 0.21	0.2683	0.09	0.6450	0.25	0.1815	0.06	0.7686
LDL-C (mg/dl)	31	− 0.34	0.0578	− 0.10	0.5803	− 0.26	0.1580	− 0.04	0.8361	0.29	0.1085	0.05	0.7960
HDL-C (mg/dl)	31	− 0.02	0.9027	− 0.05	0.7925	0.18	0.3306	0.29	0.1200	− 0.20	0.2831	− 0.14	0.4533
TG (mg/dl)	31	− 0.03	0.8745	0.16	0.3967	− 0.15	0.4183	0.27	0.1436	0.31	0.0857	0.14	0.4475
Salivary testosterone (pg/ml)	31	− 0.44	0.0138*	− 0.37	0.0424*	− 0.06	0.7338	0.00	0.9888	0.33	0.0740	0.07	0.7019
	28	0.11	0.5725	− 0.05	0.8197	− 0.16	0.4145	0.01	0.9526	0.01	0.9438	0.41	0.0312*
TNF-α (pg/ml)	31	− 0.26	0.1664	− 0.13	0.4909	− 0.20	0.2910	− 0.15	0.4318	0.54	0.0018*	N.A	N.A
	28	0.35	0.0668	0.10	0.6007	0.00	1.0000	0.19	0.3240	0.12	0.5471	N.A	N.A
IL-6 (pg/ml)	31	− 0.24	0.1976	− 0.07	0.7172	− 0.10	0.5991	0.05	0.3935	N.A	N.A	–	–
	28	0.01	0.9801	− 0.06	0.7510	− 0.20	0.3046	0.25	0.2006	N.A	N.A	–	–
IL-1β (pg/ml)	31	0.06	0.7342	0.08	0.6759	0.06	0.7680	N.A	N.A	–	–	–	–
	28	− 0.14	0.4634	− 0.26	0.2551	0.01	0.9643	N.A	N.A	–	–	–	–

TT, total testosterone; FAI, free androgen index; SHGB, sex hormone-binding globulin; DHEA-S, dehydroepiandrosterone sulfate; HOMA-IR, Homeostatic Model Assessment of Insulin Resistance; TC, total cholesterol; HDL-C, high-density lipoprotein cholesterol; LDL-C, low-density lipoprotein cholesterol; TG, triglycerides; TNF-α, tumor necrosis factor α; IL-6, interleukin 6; IL-1β, interleukin 1β; N.A., not applicable

^*^Statistically significant correlations

GI was positively correlated with PI (r_s_ = 0.61 and r_s_ = 0.66, for the PCOS group and the control group, respectively, *p* < 0.001), and negatively correlated with salivary testosterone levels in the PCOS group (r_s_ = − 0.44, *p* = 0.0138). BOP% was positively correlated with PI (r_s_ = 0.60 and r_s_ = 0.79, for the PCOS group and the control group, respectively, *p* < 0.001), BMI in the control group (r_s_ = 0.40, *p* = 0.0368), fasting insulin (r_s_ = 0.36, *p* = 0.0469), HOMA-IR in the study group (r_s_ = 0.48, *p* = 0.0068), and negatively correlated with salivary testosterone levels in the PCOS group (r_s_ = − 0.37, *p* = 0.0424). PD was positively correlated with BMI in the control group (r_s_ = 0.37, *p* = 0.0497).

Salivary testosterone was positively correlated with TNF-α in the control group (r_s_ = 0.41, *p* = 0.0321). TNF-α was positively correlated with IL-6 (r_s_ = 0.54, *p* = 0.0018).

TT was positively correlated with IL-6, while FAI was positively correlated with TNF-α (r_s_ = 0.37, *p* = 0.040, and r_s_ = 0.36, *p* = 0.0491, respectively).

## Discussion

This study aimed to assess the metabolic and hormonal factors affecting the gingival health of young females suffering from PCOS. Oral hygiene was the most critical determinant of gingiva's condition with statistically significant correlations between PI, GI and BOP%. It is a well-known phenomenon that confirms dental plaque's role in periodontal diseases' etiology [25].

Several studies demonstrated an association between obesity and periodontal problems in adults and adolescents [30–38]. Dursun et al. proved that the young obese women showed higher gingival index and gingival bleeding index with no significant difference in probing depth, and clinical attachment level, than the lean women with similar plaque index. Clinical periodontal indices showed significant correlations with BMI, insulin, lipid levels, and oxidant status markers [34]. Diet induced obesity in mice has been shown to decrease the immune system’s ability to respond to periodontal pathogens [36]. Positive correlations between BMI and two periodontal indices in the control group seem to confirm other authors’ claims. At the same time, no significant correlations between BMI and proinflammatory cytokines in saliva were detected in the present study. Similarly, in our previous study, we did not observe the significant differences between serum levels of CRP, TNF-α, IL-1, and IL-6 of girls with PCOS and normal weight compared to the group with overweight and obesity [39]. Although adipose tissue is a source of TNF-α, IL-1, and IL-6 [37], the level of inflammatory markers in the body fluids may depend on other factors, such as glucose tolerance [40] and diet [39]. Since excessive weight is a common trait of PCOS, it is difficult to distinguish between the impact of the metabolic complications of being overweight and the effects of the disease itself [40, 41]. Mohlig et al. concluded that PCOS per se is not associated with increased chronic inflammation in the body. In their study, neither CRP nor IL-6 were significantly elevated in lean or obese PCOS women compared with age-matched lean or obese controls. Variables of body composition (BMI, waist to hip ratio) and insulin resistance were correlated with CRP or IL-6, while parameters of hyperandogenism were not [41]. It argues with the conclusions of Dursun et al., who observed an increased susceptibility for gingivitis and a local/periodontal prooxidative state in lean women with PCOS compared with healthy controls [8].

Regarding the impact of hyperandrogenism on the gingival health, sex hormones have been shown to have a profound influence on the function of inflammatory cells and the secretion and activation of a range of inflammatory mediators [42]. In the study by Daltaban et al., testosterone deficiency negatively influenced periodontal disease in men with hypergonadotropic hypogonadism [43]. On the other hand, in the study by Brusca et al., anabolic androgenic steroids users had a significantly higher prevalence of severe periodontitis, more significant gingival inflammation, and clinical attachment loss of ≥ 3 mm, which was attributed to the considerably higher proportions of *A. actinomycetemcomitans*, *P. gingivalis, P. intermedia*, and Candida species as compared to controls [44]. Animal studies showed that sub‐physiological levels of testosterone were associated with increased gingival concentrations of IL‐1β, IL‐6, and TNF‐α [45–47], suggesting that adequate levels of testosterone are necessary to maintain gingival health. Testosterone and androgen receptors turned out to play an essential role in the periodontal repair process in female rats [48, 49]. In our study, salivary testosterone was negatively correlated with GI and BOP% in the PCOS group. On the other hand, TT level was positively correlated with salivary IL-6, FAI was positively correlated with TNF-α, while in the control group, salivary testosterone was positively correlated with TNF-α. These correlations can be probably attributed to the metabolic impact of androgen excess in PCOS. As reminded by Sanchez-Garrido et al. hyperandrogenism in women has a detrimental effect on different metabolic tissues, including the adipose tissue, liver, pancreas, and skeletal muscles, increasing adiposity and reducing insulin sensitivity [18]. In the study by Özçaka et al., the group of women with hyperandrogenism exhibited significantly higher saliva TNF‐α concentrations than the control group, independent of the presence of gingivitis [5].

In our study, involving young subjects with good oral health maintenance, indices of periodontal health of the group with endocrinopathy and healthy controls were similar. At the same time, patients with PCOS had significantly higher proinflammatory cytokines than their healthy age-mates. In order to explain this phenomenon, we must emphasize that the whole saliva is composed of the salivary glands' secretions, gingival crevicular fluid, and mucosal transudations, derived from the blood [50]. In our study, salivary proinflammatory cytokines did not correlate with periodontal parameters. Thus, it is possible that in the examined group of PCOS patients, systemic inflammation dominated over the local secretion of cytokines, which resulted in higher salivary IL‐1β, IL‐6, and TNF‐α levels.

Similar conclusions were drawn by Redman et al., who found that salivary and serum CRP levels remained correlated in patients with rheumatoid arthritis or osteoarthritis independent of the periodontal condition. They suggested that elevation in salivary CRP due to periodontitis was overshadowed by differences among examined patients in factors such as the presence of arthritis, the use of anti-inflammatory medications, and the extent of adipose tissue [51]. Riis et al. reported opposite results in healthy adolescent girls aged 11–17. Their findings indicate that variation in salivary cytokine levels reflects the local inflammatory activity of the oral mucosa rather than systemic inflammation [52]. These conflicting data can be explained by the differences in general health, oral health and systemic inflammatory status of examined populations.

As far as other metabolic syndrome components are concerned, there is evidence in the literature that the anomaly of lipid metabolism in the blood may affect periodontal disease progress [53, 54]. Animal studies have shown that dyslipidemia predisposes the host to oral infection by impairing proper immune response to bacteria challenge [55, 56]. On the other hand, the periodontal inflammation induced by exposure to periodontal pathogens (*T. denticola*,* P. gingivalis*,* T. forsythia*,* and P. intermedia*) induced dyslipidemia by lowering serum HDL-C level [57]. In our study, the lipid profile of female with PCOS did not affect gingival health. However, only a few subjects had dyslipidemia.

Insulin resistance has also been linked to increased susceptibility to infections and altered oral microflora composition [58]. There is a considerable body of evidence suggesting the existence of a two-way relationship between diabetes and periodontal disease, with diabetes increasing the risk for periodontitis, and periodontal inflammation negatively affecting glycaemic control [59–61]. Our subjects did not present abnormal glucose levels, but many had elevated insulin resistance index. A significant moderate correlation between HOMA-IR and percentage of bleeding upon probing sites and weak correlation between fasting insulin level and BOP% confirm the association between insulin resistance and gingivitis.

Finally, there is increasing evidence in the literature that psychosocial stress, depression, and anxiety might be significant risk factors of periodontal disease [62]. Ishisaka et al. observed an association between levels of the stress‐related steroid hormones (cortisol and DHEA-S), as well as their ratio and periodontitis in elderly subjects [63]. Glucocorticoids exert suppressive actions on the immune and inflammatory responses, which may lead to the establishment of periodontal infection and result in destructive periodontitis [62]. DHEA-S might block the development of the pathological processes potentiated by a prolonged increase in cortisol secretion [63]. Since PCOS is associated with psychological stress and mental problems [1–3], it could be linked to periodontal disease through the pathway mentioned above. As previously proved, emotional stress, anxiety and depression could also result in elevated levels of proinflammatory cytokines in saliva and blood plasma [64]. However, our study did not reveal significant correlations between periodontal health, stress-related hormone levels and salivary concentrations of IL-1β, TNF-α and IL-6.

The study has some limitations that should be taken into account during the interpretation of the obtained results. Firstly, all subjects presented good oral health maintenance, so the ranges of GI, BOP% and PD were relatively narrow and possible differences between groups were difficult to capture. Secondly, due to ethical concerns, the control group was recruited without blood tests. Thus, neither could we assess the metabolic and hormonal profile of healthy subjects nor compare both groups’ inflammatory status based on serum proinflammatory cytokines. Furthermore, we did not radiographically assess alveolar bone loss and evaluate the condition of periodontium based on GI, BOP%, and PD.

## Conclusions

The results of the study seem to confirm our initial hypothesis and suggest that young women with PCOS and good oral health maintenance do not differ from healthy controls concerning gingival indices. The gingival health of the examined population was associated primarily with oral hygiene and, to a lesser extent, with hormonal and metabolic profile. Despite similar periodontal parameters, the study group had significantly higher levels of proinflammatory cytokines in saliva, which may be the manifestation of the chronic low-grade systemic inflammation associated with PCOS.

## Data Availability

The data that support the findings of this study are available on reasonable request from the corresponding author.

## Abbreviations

PCOS: Polycystic Ovary Syndrome
BMI: Body mass index
PI: Plaque index
GI: Gingival index
BOP%: Bleeding on probing score
PD: Probing depth
FSH: Follicle-stimulating hormone
LH: Luteinizing hormone
TT: Total testosterone
FAI: Free androgen index
SHGB: Sex hormone-binding globulin
DHEA-S: Dehydroepiandrosterone sulfate
HOMA-IR: Homeostatic Model Assessment of Insulin Resistance
TC: Total cholesterol
HDL-C: High-density lipoprotein cholesterol
LDL-C: Low-density lipoprotein cholesterol
TG: Triglycerides
TNF-α: Tumor necrosis factor α
IL-6: Interleukin 6
IL-1β: Interleukin 1β
